# Policy-Based Holistic Application Management with BPMN and TOSCA

**DOI:** 10.1007/s42979-022-01616-w

**Published:** 2023-02-23

**Authors:** Domenico Calcaterra, Orazio Tomarchio

**Affiliations:** Department of Electrical, Electronic and Computer Engineering, University of Catania, V.le A. Doria 6, 95125 Catania, Italy

**Keywords:** Cloud management automation, Holistic application management, Policies, TOSCA, BPMN

## Abstract

With the wide adoption of cloud computing across technology industries and research institutions, an ever-growing interest in cloud orchestration frameworks has emerged over the past few years. These orchestration frameworks enable the automated provisioning and decommissioning of cloud applications in a timely and efficient manner, but they offer limited or no support for application management. While management functionalities, such as configuring, monitoring and scaling single components, can be directly covered by cloud providers and configuration management tools, holistic management features, such as backing up, testing and updating multiple components, cannot be automated using these approaches. In this paper, we propose a concept to automatically generate executable holistic management workflows based on the TOSCA standard. The practical feasibility of the approach is validated through a prototype implementation and a case study.

## Introduction

As cloud growth has accelerated over the course of the past few years, more and more applications are deployed on a cloud environment with the result of a larger distribution of services across the internet [13]. In the aftermath of the COVID-19 pandemic, to remain competitive in today’s increasingly digital world, organisations have to make strategic decisions about their cloud migration, cloud architecture, effective tooling and cost management [16]. Despite a wide variety of cloud providers and services [14], an intrinsic complexity resides in application deployment and management which is considered to be a draining, error-prone and time-consuming process [6]. In this respect, cloud orchestration tools have increased their popularity in recent years, becoming a main topic for cloud research [2, 5].

Cloud orchestration regards various complex operations to select, configure, deploy, monitor and control resources or services over different cloud solutions in an automated fashion [32]. There exist different approaches to cloud orchestration [4], ranging from commercial orchestration platforms to cloud-agnostic tools [11, 21, 22]. Nonetheless, they can vary greatly in configuration languages, complexity and compatibility, posing great challenges for those who lack cloud-specific skills or knowledge on how to efficiently use the available set of automation tools.

While all these approaches fully support deployment automation, they only provide limited or no management automation. In accordance with current practice, management features are integrated as infrastructure services resulting in additional management code, human intervention and vendor lock-in [28]. Furthermore, holistic management functionalities affecting multiple components of an application, e.g., backing up the application state, testing and/or updating application components, are mostly unsupported. Especially in DevOps scenarios, where applications change frequently, a complete lack of holistic management can rapidly lead to faulty deployments. Therefore, modern orchestration frameworks should tackle these issues while providing their management tasks.

In this paper, we extend the work presented in Calcaterra and Tomarchio [7] where we proposed a concept for the automated generation of holistic management workflows based on TOSCA application models and policies. In this work, we carry out a more in-depth analysis of the approach and discuss a brand new management operation. In this regard, we improve our previous work by providing integration with update mechanisms for a variety of application components. The new management feature is supported via new interface types, node types and policy types included in our TOSCA extension. We also revisited the case study to validate the additional management operation.

The remainder of the paper is structured as follows. “Background” provides a background of the concepts used in this work. “Motivation” motivates the need for holistic management automation. “Conceptual Overview” presents the core ideas for management automation. “Policy-Based Application Management” delves into the proposed approach to automatically generate management workflows from TOSCA-based application models. “Prototype Validation” and “Case Study” discuss a prototype implementation and a case-study showing the potential of the proposed idea, respectively. Related work is addressed in “Related Work”. Concluding remarks and future directions are debated in “Conclusion”.

## Background

In this section, we first cover the basics of deployment and management automation and then introduce the TOSCA specification.

### Deployment and Management Automation

Both industry and academia have given more and more attention to orchestration and management frameworks over the past few years [29]. Most big industry players have also developed Cloud Management Platforms (CMP) to automate cloud provisioning and offer lifecycle management of cloud applications. These commercial platforms are generally neither open to the community nor portable across third-party providers.

There are a few tools sharing similarities with cloud orchestrators. Configuration management tools, such as Ansible, Chef, Puppet and Salt, automate the development, delivery, testing and maintenance throughout the software lifecycle. Infrastructure as Code (IaC) tools have also appeared to change, configure and automate infrastructure. Terraform is one of the most notable IaC open-source solutions. All of these technologies use either *declarative* or *imperative* models to automate application deployment. While declarative models describe the application structure and its desired state from which all the required deployment tasks are automatically derived, imperative models define the deployment tasks to be executed, the control flow and the data flow between them [15].

Despite being highly intuitive and reusable, declarative models have their limitations. Deployment systems can directly infer all tasks to be executed from the models, but they can rarely customise tasks or alter their execution order. Imperative models are necessary when it comes to complex application deployments with customised tasks. Workflows languages, such as BPMN [26] and BPEL [23], are typical examples of imperative technologies. The other side of the coin is that imperative models require technical expertise and are frequently outdated as compared to declarative models.

While the available technologies support automated deployments over multiple environments, they only provide limited automated management [28]. Cloud providers typically offer management features for the hosted components only, resulting in the need for single management features to be orchestrated when it comes to multiple providers. Holistic management of multiple components distributed across different environments is mostly unsupported as well. Typical holistic management features include but are not limited to component backup, testing, update, etc., which require single management features to be orchestrated (by a workflow, for instance).

### The TOSCA Specification

Specification languages to describe cloud applications simplify the orchestration process and promote interoperability across different providers. TOSCA [24] represents a notable contribution to cloud standardisation, since it allows to describe applications and their lifecycle management in a vendor- and technology-independent fashion [3].

TOSCA describes the structure of a cloud application as a *service template*, which consists of a topology template and all the types needed to build such a template. The topology template is a typed directed graph, whose nodes (called *node templates*) model the application components, and edges (called *relationship templates*) model the relations among such components. Each topology node comes with the corresponding *capabilities* and *requirements*, the *interfaces* to manage it, the *attributes* and *properties* it features, the software *artifacts* it uses and the *policies* applied to it.

TOSCA supports application deployment and management in two different flavours: *imperative processing* and *declarative processing*. The imperative processing requires that management logic is contained in the Cloud Service Archive (CSAR), which stores all software artifacts needed to provision and manage the application. Management plans imperatively orchestrate low-level management operations that are provided either by the application components or by publicly accessible services. Management plans are typically implemented using workflow languages (e.g., BPMN, BPEL). The declarative processing shifts management logic from plans to runtime. TOSCA runtimes automatically infer the corresponding logic by interpreting the application topology template. Management functionalities depend on the corresponding runtime, which is not standardised by the TOSCA specification.

TOSCA Simple Profile [25] is an isomorphic rendering of a subset of the TOSCA specification in the YAML language. It provides a more accessible syntax as well as a more concise and incremental expressiveness to speed up the adoption of TOSCA to describe portable cloud applications. TOSCA Simple Profile defines a few normative workflows to operate a topology and specifies how they are declaratively generated: deploy, undeploy, scaling workflows and auto-healing workflows. Imperative workflows can be used for complex use cases where declarative workflows do not suffice. Nevertheless, they provide less reusability as they are topology specific rather than being dynamically generated based on the topology content. The work described in this paper heavily grounds on the TOSCA Simple Profile.

## Motivation

As discussed in “Deployment and Management Automation”, current approaches support automated deployment over multiple environments, but they only provide limited automated management which demands orchestration whenever multiple cloud providers, services and components are involved. By contrast, automated holistic management is mostly unsupported, unless custom implementations come into play.

**Fig. 1:**
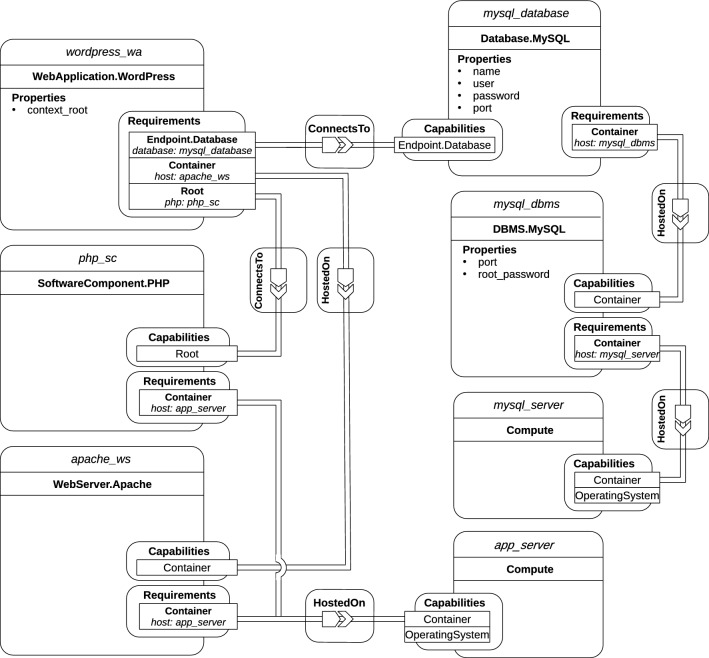
TOSCA application model of a WordPress scenario

Figure 1 illustrates a declarative application model describing a typical TOSCA-based cloud application. To have a correct application deployment, it is crucial to ascertain whether or not it is successful from both a technical and business standpoint. Testing is the main way to reach the goal. In case of multi-cloud applications, it might be necessary for application components to communicate with each other. For instance, depending on the testing, SSH connections (e.g. *Compute* nodes), HTTP connections (e.g. *WordPress* and *Apache* nodes) or even SQL connections (e.g. *DBMS* and *Database* nodes) might need to be established. Since massive expertise is required for testing, automated test generation is important to ensure that all components and communication among them work as expected. In case of a web application, it is also important to copy current data on a regular basis. To back up the Database in our scenario, it is required to either use the backup feature of the underlying DBMS or establish a direct connection to the database and execute a query to retrieve all data. However, additional technology and domain-specific logic need to be orchestrated and executed in the correct order.

Beside testing and backup, additional management functionalities could be considered. For instance, throughout a typical application life-cycle, application components might need updates of different kinds. By way of example, let us consider a DBMS component and a Compute node. For the former, a couple of update types might occur: configuration update (e.g., port number change) and version update. For the latter, update operations might include package update and user creation and/or update. Even in this case, since these update operations demand custom logic and considerable expertise, a mechanism to enable automated updates on application components is needed.

In general, automating application management can be a major challenge when management features must be implemented manually, since manual implementations require extensive domain-specific expertise and are error-prone, time consuming and frequently outdated. In this work we propose an approach for the automatic generation of holistic management workflows based on TOSCA application models and policies.

## Conceptual Overview

**Fig. 2:**
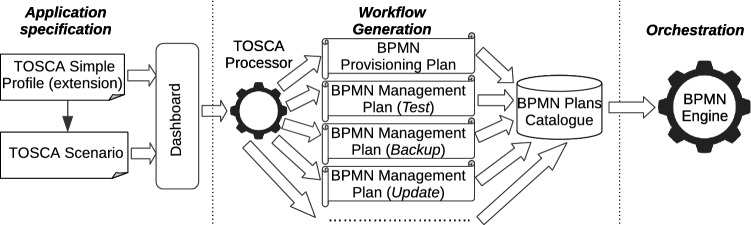
Overview of the automated generation of application management workflows

In this section, we provide a bird’s-eye view of the methodology to automatically generate executable management workflows from TOSCA application models. The approach is depicted in Fig. 2.

The overall process consists of three distinct sequential phases, namely *Application Specification*, *Workflow Generation* and *Orchestration*. The process commences with the *Application Specification* phase, where the application architect is responsible for modelling and submitting a TOSCA application model which is specified based on *interface types*, *node types* and *policy types* included in a TOSCA Simple Profile extension. All these types contributes to the definition of management interfaces and policies for different management features, using a general-to-specific pattern. In fact, considering a general management feature (e.g. testing), general interface types and policy types are defined for such a feature which get to be extended for specific node types (e.g. Database). Also, *Managed node types* are defined by extending basic TOSCA node types based on all available management interfaces. A thorough discussion of interface types, policy types and managed node types is provided in “Application Specification”.

The submitted input triggers the *Workflow Generation* phase, which in turn consists of the transformation of a TOSCA application model into different workflow plans, namely provisioning and management plans (e.g. backup, testing, update). The provisioning plan is generated based on node dependencies, whereas management plans are generated based on policies and management interface operations defined on nodes. Depending on the specific management feature, different strategies (e.g. parallel, sequential) can also be used for the automated generation of management plans. The TOSCA Processor component is in charge of parsing a TOSCA application model, generating provisioning and management plans for it and validating them. Ultimately, in the *Orchestration* phase, a workflow engine executes such plans. The generation of management plans and the framework enacting the entire process are investigated in “Management Workflow Generation” and “Prototype Validation”, respectively.

## Policy-Based Application Management

In this section, we fully explore the proposed approach to automatically generate management workflows from TOSCA-based application models. The main steps of the entire process are covered below. In particular, three management features are taken into consideration with reference to application specification and workflow generation: *backup*, *testing* and *update*.

### Application Specification

One of the main strengths of this work is the development of a standards-based approach for the description of application topology and management, which leverages the TOSCA standard. TOSCA application models are described by means of a TOSCA Simple Profile extension. In fact, while normative interface types and node types provide sufficient deployment capabilities, the current version of the TOSCA standard is not well equipped with management features. In order to fill this gap, we provide application models with additional management information to enable the automated generation of holistic management workflows.

Application models are enriched with specific *interface types*, which get to be extended depending on a combination of management features and node type categories, *node types*, which extend normative node types and include these management interfaces, and *policy types*, which define the operations to execute on the targeted node types based on management features. The complete set of extended types is fully TOSCA-compliant, since it is valid according to the grammar and rules defined in the standard, and can be further enriched with additional management features. More details about these types are provided in the following subsections.

#### Interface Types

Although the TOSCA Simple Profile specification includes two normative interface types, i.e., *Standard* and *Configure*, for component lifecycle and configuration respectively, there is a lack of support for management features. As a result, we extended the standard specification by defining management-oriented interface types.

**Fig. 3:**
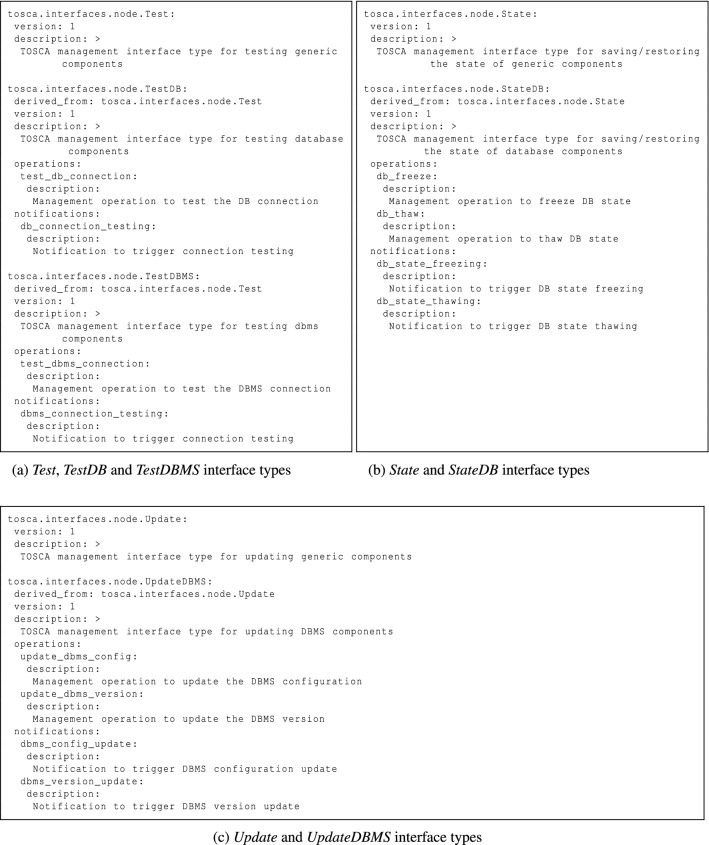
Illustrative interface types for Testing, Backup and Update features

For the sake of clarity, Fig. 3 shows a few exemplifying interface type definitions included in our TOSCA Simple Profile extension. As mentioned in “Conceptual Overview”, given a specific management feature, interface types are defined via a general-to-specific pattern. As we can see in Fig. 3a, two interface types are present: a general interface type (*Test*), which is defined for testing purposes, and a specific interface type (*TestDB*), which is an extension of the former for testing database components. Figure 3b shows two interface type definitions: a general interface type for backup purposes and a specific interface type for backing up database components. Similarly, Fig. 3c illustrates two interface type definitions: a general interface type for update purposes and a specific interface type for updating DBMS components.

#### Node Types

**Fig. 4:**
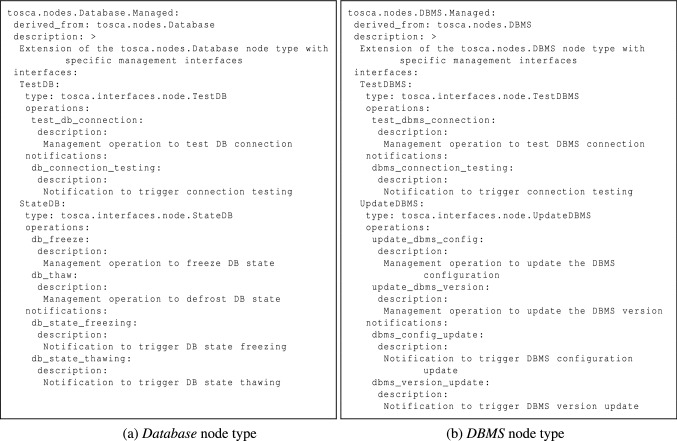
Illustrative node types for *Testing*, *Backup* and *Update* features

All these additional interface types must be included in node type definitions, which is why we also extended the normative node type hierarchy to support management features. By way of illustration, Fig. 4 depicts two node type definitions included in our TOSCA Simple Profile extension. In particular, as we can see in Fig. 4a, the *Database.Managed* node type extends the normative *Database* node type by adding *TestDB* and *StateDB* interfaces (see Fig. 3) for testing and backup purposes. By contrast, Fig. 4b shows the *DBMS.Managed* node type, which extends the normative *DBMS* node type by adding *TestDBMS* and *UpdateDBMS* interfaces (see Fig. 3) for testing and update purposes.

#### Policy Types

**Fig. 5:**
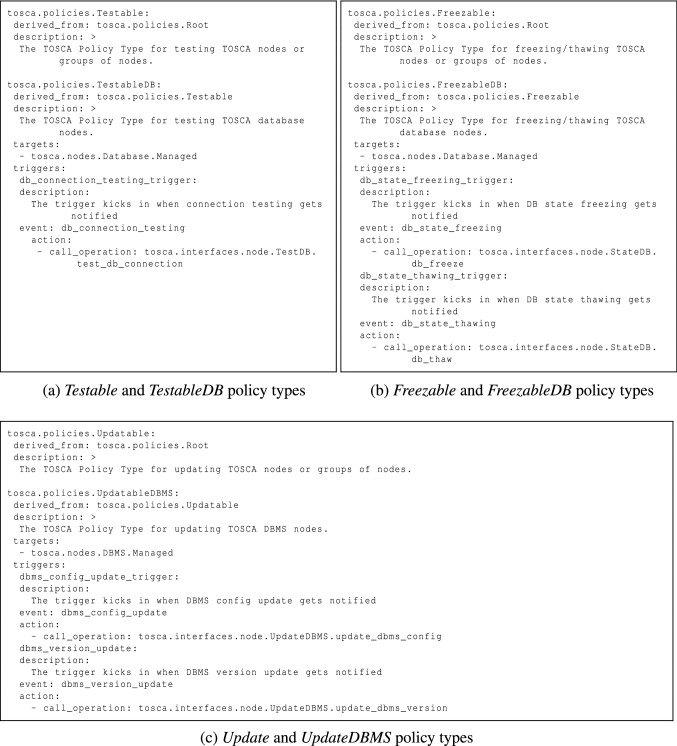
Illustrative policy types for *Testing*, *Backup* and *Update* features

Policy types are also defined to specify the targeted node types and the actions to perform in relation to management features, when specific events are triggered. Specifically, policy triggers are linked to notification events from the targeted node types’ management interfaces. Similar to interface types, policy types are defined via a general-to-specific pattern as well. As we can see in Fig. 5, a few exemplary policy types are present. Figure 5a depicts the *Testable* general policy type for testing purposes and the *TestableDB* specific policy type for testing database components. Figure 5b illustrates the *Freezable* general policy type for backup purposes and the *FreezableDB* specific policy type for backing up database components. Lastly, Fig. 5c shows the *Updatable* general policy type for update purposes and the *UpdatableDBMS* specific policy type for updating DBMS components.

### Management Workflow Generation

As mentioned in “Conceptual Overview”, the application architect models a TOSCA application model according to interface types, node types and policy types included in a TOSCA Simple Profile extension. As a result, the application model is automatically enriched with management capabilities, which the TOSCA Processor (see Fig. 2) leverages to generate management workflows based on policies and interface operations defined on nodes.

Policies are of paramount importance, as they specify the targeted nodes and the actions triggered depending on management features. In addition, the strategy for the generation of management workflows depends on management features. The following subsections detail two different strategies, namely parallel strategy and sequential strategy, and how they relate to each other with respect to three management features: backup, testing and update.

#### Backup Feature

**Fig. 6:**
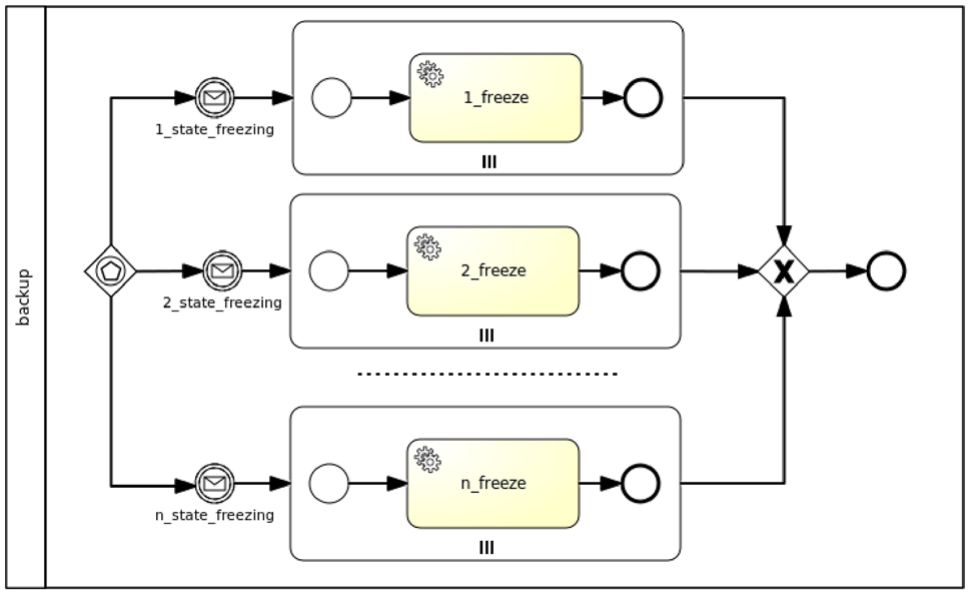
Parallel strategy for Backup workflows

In case of the *backup* feature a parallel strategy is adopted, since every stateful component can be backed up independently. In fact, although some components can either directly or indirectly list different components as node requirements, the backup of their state would not necessarily be affected as opposed to other management features (i.e. *testing* and *update*). The parallel generation of Backup workflows works as shown in Fig. 6.

In general, considering more than one *Freezable* policies (see Fig. 5b), a new workflow is generated where more than one paths can trigger the workflow instantiation by means of an event-based gateway. Each path consists of a message intermediate event, which is linked to a notification event from the targeted nodes’ *State* interface (see Fig. 3b), and a parallel multi-instance subprocess, whose number of instances depends on the number of target nodes the policy is applied to. A service task in the subprocess executes the action of the policy trigger, which enacts the actual backup.

#### Testing Feature

**Fig. 7:**
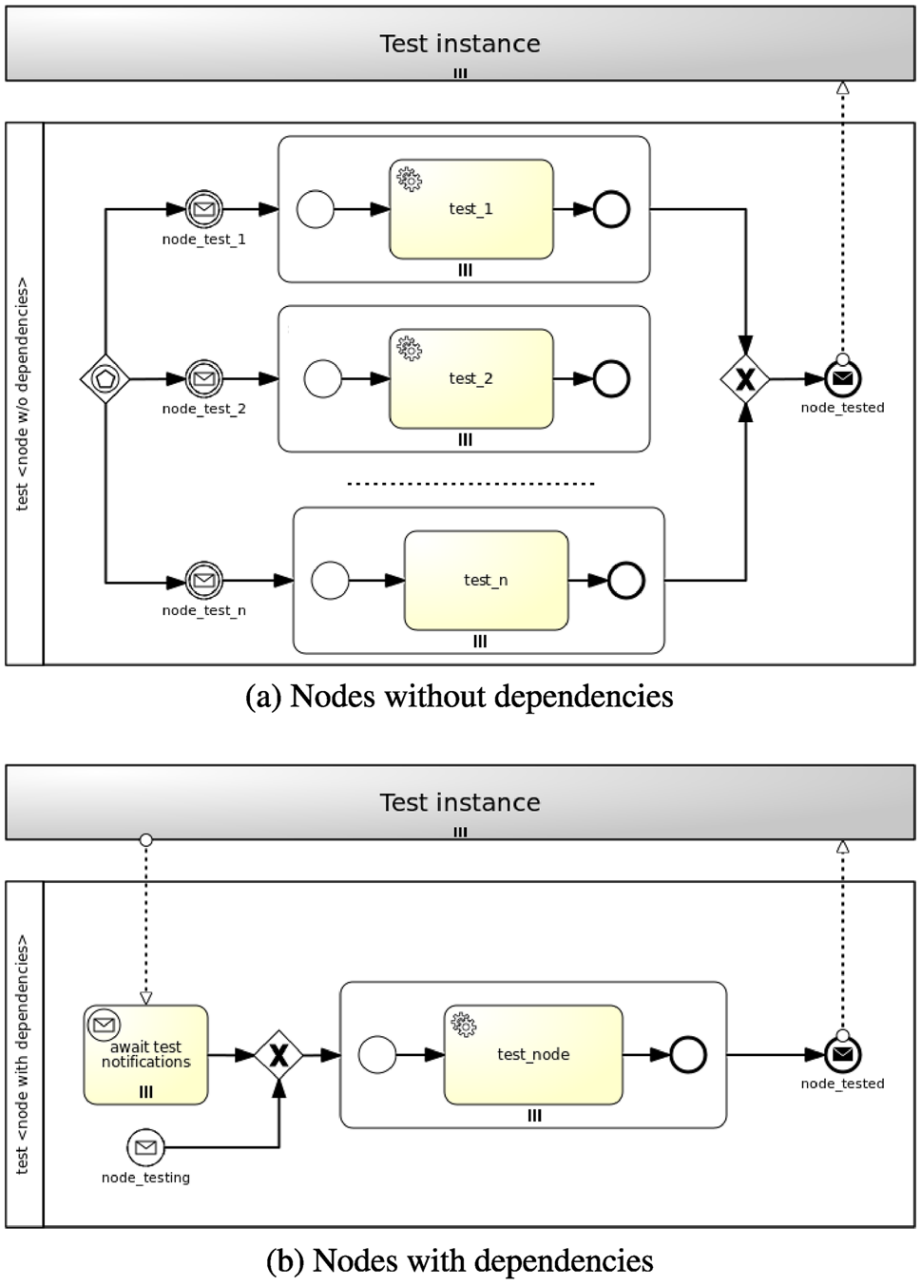
Parallel and sequential strategies for Testing workflows

In case of the *testing* feature two strategies are viable: (a) parallel strategy and (b) sequential strategy. Components can be either tested in isolation with the parallel strategy or tested consecutively following node dependencies with the sequential strategy. It is worthy of note that all node dependencies can be automatically determined based on node requirements and relationships in a TOSCA application model [8, 9]. Figure 7 shows how Testing workflows are generated. Firstly, a workflow is generated for each node type. Secondly, a distinction is made between nodes without dependencies and nodes with dependencies. The former are tested in isolation, while the latter can be either tested in isolation or tested sequentially according to node dependencies.

In the first case (see Fig. 7a), considering more than one testing operations, a new workflow is generated where more than one paths can trigger the workflow instantiation by means of an event-based gateway. Each path consists of a message intermediate event, which is linked to a notification event from the targeted nodes’ *Test* interface (see Fig. 3a), and a parallel multi-instance subprocess, whose number of instances depends on the number of target nodes the policy is applied to. A service task in the subprocess executes the action of the policy trigger, which enacts the actual testing. Finally, a message end event marks the end of the workflow.

In the second case (see Fig. 7b), a new workflow is generated where either a message start event, coming from the targeted nodes’ *Test* interface notifications, or a parallel multi-instance receive task, waiting for notifications from node requirements under test, triggers the workflow instantiation. A parallel multi-instance subprocess is then activated depending on the number of target nodes the policy is applied to. A service task in the subprocess executes the action of the policy trigger enacting the actual testing. Finally, a message end event marks the end of the workflow.

#### Update Feature

**Fig. 8:**
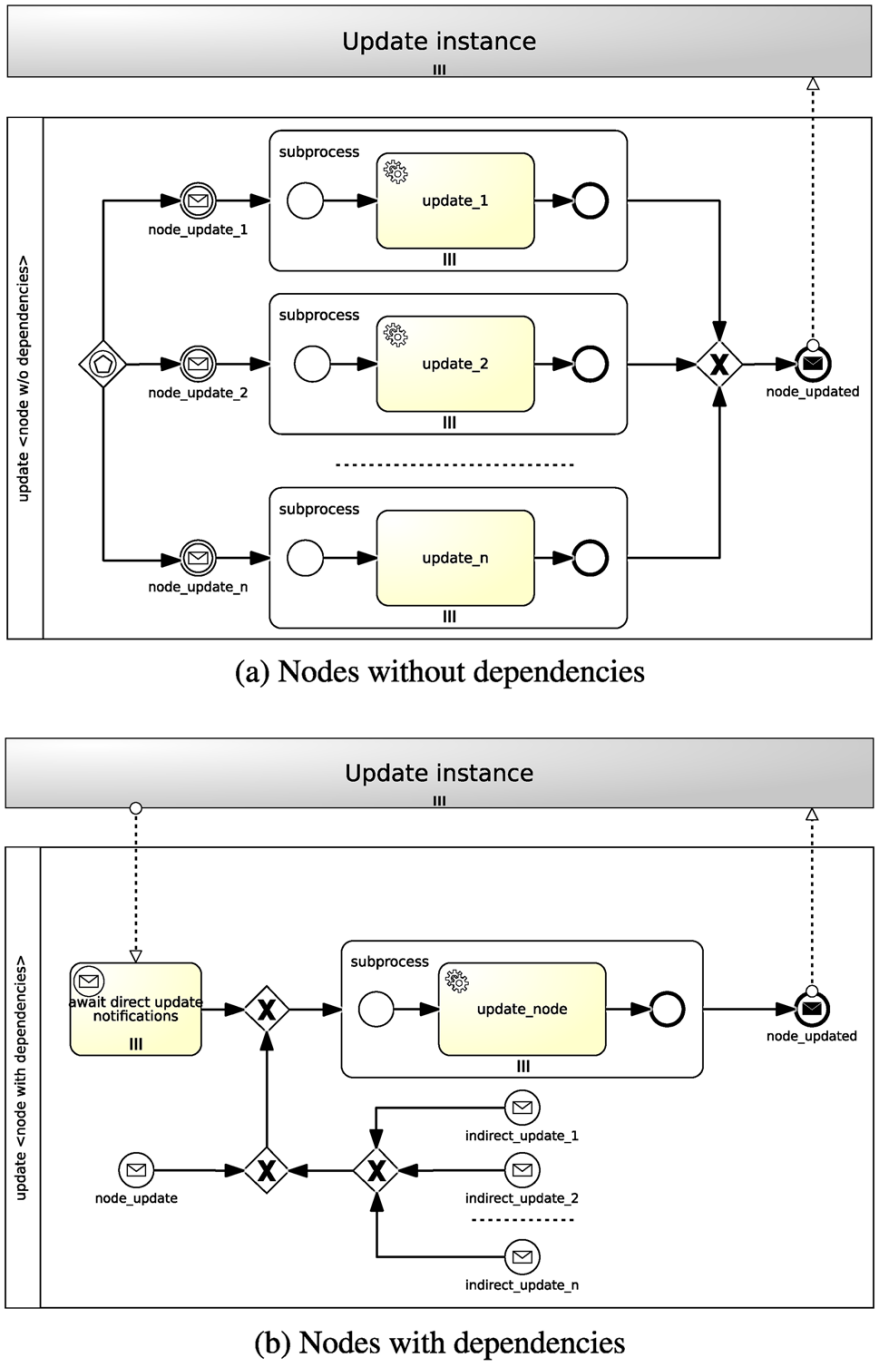
Parallel and sequential strategies for Update workflows

Similar to the testing feature, both parallel strategy and sequential strategy can be applied for the update feature as well. The parallel strategy allows to update components in isolation, whereas the sequential strategy enables to update them consecutively according to node dependencies. Figure 8 shows how Update workflows are generated.

A workflow is generated for each and every node type. We must make a sharp distinction between nodes without dependencies and nodes with dependencies. While the former can be updated in isolation, the latter can be updated either in isolation or sequentially based on direct and/or indirect dependencies. *Direct dependencies* originate from node requirements and relationships and reflect the dependency graph. These are the same dependencies from which deployment workflows are generated too. *Indirect dependencies* refer to transitive dependencies which are induced by nodes acting as direct dependencies. For the sake of clarity, with reference to the motivation scenario in “Motivation”, the *wordpress_wa* node includes direct dependencies towards three other nodes: *apache_ws*, *mysql_database* and *php_sc*. In addition, it also features an indirect dependency towards the *mysql_dbms* node, since a DBMS node update can have an impact on a WordPress node. For instance, a configuration update on a DBMS node (e.g., changing the DBMS port) usually leads to a configuration update on a WordPress node.

In the absence of node dependencies of any sort (see Fig. 8a), given more than one update operations, a new workflow is generated where more than one paths can trigger the workflow instantiation via an event-based gateway. Each path includes a message intermediate event, which is linked to a notification event from the targeted nodes’ *Update* interface (see Fig. 3c), and a parallel multi-instance subprocess, whose number of instances depends on the number of target nodes the policy is applied to. A service task in the subprocess executes the update action of the policy trigger. Ultimately, a message end event marks the end of the workflow.

When node dependencies are present (see Fig. 8b), a new workflow is generated where one of a parallel multi-instance receive task, waiting for notifications from direct node dependencies, a message start event, coming from the targeted nodes’ *Update* interface notifications, and one of the targeted nodes’ indirect dependencies triggers the workflow instantiation. A parallel multi-instance subprocess is then activated depending on the number of target nodes the policy is applied to. A service task in the subprocess executes the update action of the policy trigger. In conclusion, a message end event marks the end of the workflow.

## Prototype Validation

To validate our approach, we implemented a prototype based on the open-source TORCH [30], which is a TOSCA-based framework for the deployment and orchestration of VM-based and container-based applications on top of different cloud providers. TORCH takes a TOSCA application model as input and turns it into an equivalent BPMN workflow and dataflow model, which a BPMN engine leverages to enforce the operations specified in the model.

**Fig. 9:**
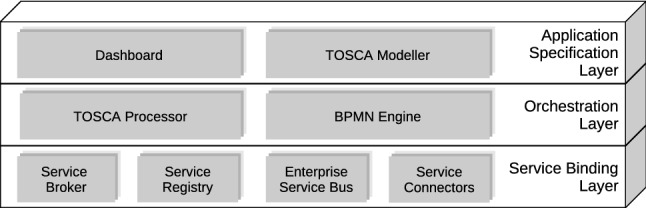
TORCH-framework architecture

Figure 9 shows the multi-layered framework architecture of TORCH. The *Application Specification Layer* consists of two components: the Dashboard, which is the front-end component for user interaction and deployment monitoring, and the TOSCA Modeller, which guides the user to sketch application requirements. The *Orchestration Layer* comprises the TOSCA Processor component, which is in charge of validating, parsing and converting TOSCA application templates into BPMN plans, and the BPMN Engine component, which is responsible for instantiating and orchestrating such BPMN plans. The *Service Binding Layer* manages the orchestration of application resources and services and consists of four components: Service Bus, Service Registry, Service Broker and Service Connectors. The Service Broker is in charge of taking care of the requests coming from the Orchestration Layer by means of the Service Bus. The Service Connectors include the logic to provision a specific resource or service, interacting with the external providers. The Service Registry is responsible for the registration and discovery of the Service Connectors.

The proposed approach for the automated generation of management workflows (see “Policy-Based Application Management”) is based on the extension of the *TOSCA Processor* (see Fig. 9), which consists of three components: *TOSCA-Parser*, *BPMN-Generator* and *BPMN-Validator*. The TOSCA-Parser provides means to load, parse and validate a TOSCA service template and create the corresponding dependency graph. The BPMN-Generator creates a BPMN plan depending on a parsed service template and its dependency graph. The BPMN-Validator validates an automatically generated plan against the BPMN specification. Further details about these components can be found in Calcaterra et al. [8, 9].

As mentioned above, the TOSCA Processor was extended to provide support for the automatic generation of holistic management workflows. A few novelties were introduced in both the TOSCA-Parser and the BPMN-Generator. With regard to the TOSCA-Parser, it was enhanced to integrate an ad-hoc parsing of node properties and artifacts, custom interfaces, interface operations and notifications, policy triggers. With respect to the BPMN-Generator, as it was originally designed to generate provisioning plans based on the TOSCA dependency graph, it was extended to support the generation of management plans based on TOSCA policies. In particular, while the original BPMN-Generator was only capable of generating workflows depending on node dependencies, the extended BPMN-Generator is capable of generating workflows even when there are no explicit node dependencies. For instance, it can support both sequential and parallel strategies for workflow generation (see “Management Workflow Generation”).

## Case Study

**Fig. 10:**
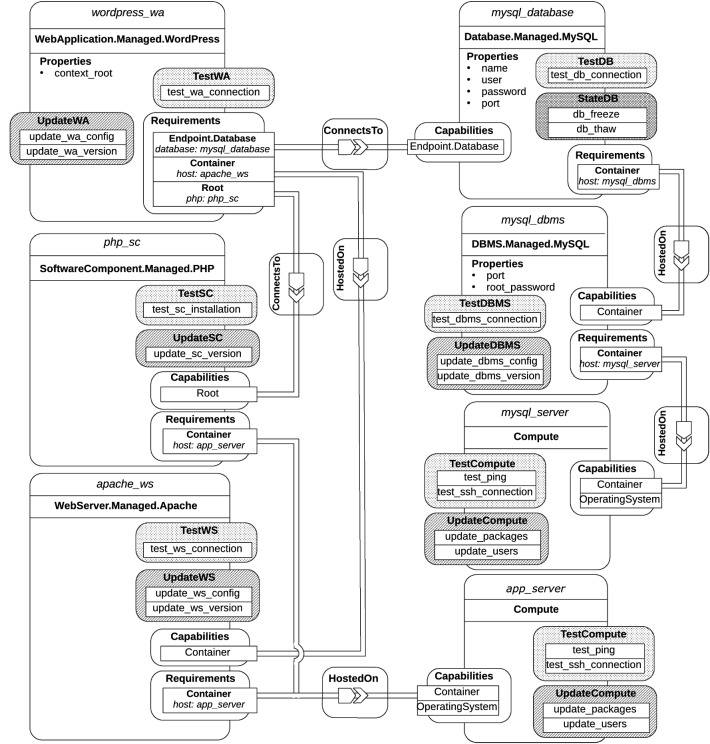
Management-oriented TOSCA application model of a WordPress scenario

Based on the motivation scenario introduced in “Motivation”, we discuss three different management features, namely *Testing*, *Backup* and *Update*. As mentioned in “Application Specification”, managed node types extend normative node types and include management interfaces containing specific management operations and their respective implementation artifacts. By applying our approach, the motivating scenario is automatically enriched with test, backup and update operations by means of feature-specific node types and policy types. The added operations are shown in Fig. 10. With reference to the backup feature, we can see that the *mysql_database* node includes the *StateDB* interface with two management operations: *db_freeze* and *db_thaw*. With regard to the testing feature, every node contains a specific interface for testing purposes. By way of illustration, we can observe that the *mysql_server* and *mysql_dbms* nodes include the *TestCompute* and *TestDBMS* interfaces, respectively. Finally, almost every node features a particular interface for updating purposes. For instance, the *app_server* and *php_sc* nodes comprehend the UpdateCompute and UpdateSC interfaces, respectively.

### Backup Feature

**Fig. 11:**
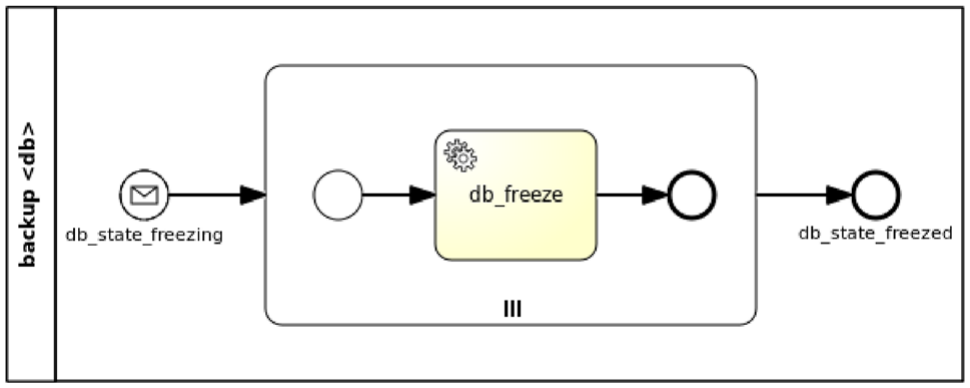
Database Backup workflow for a WordPress scenario

Based on the approach for the management workflow generation in “Management Workflow Generation”, Fig. 11 shows the Backup workflow for the *mysql_database* node in the motivation scenario. Given a *FreezableDB* policy targeting a *Database.Managed* node (see Fig. 5b), a new workflow is generated where the *db_state_freezing* message start event, coming from the StateDB interface notifications, triggers the workflow instantiation. A parallel multi-instance subprocess is then activated depending on the number of target nodes the policy is applied to. In this case there is only one target node (i.e. *mysql_database*). The *db_freeze* service task in the subprocess executes the action of the policy trigger, which enacts the actual database backup.

### Testing Feature

**Fig. 12:**
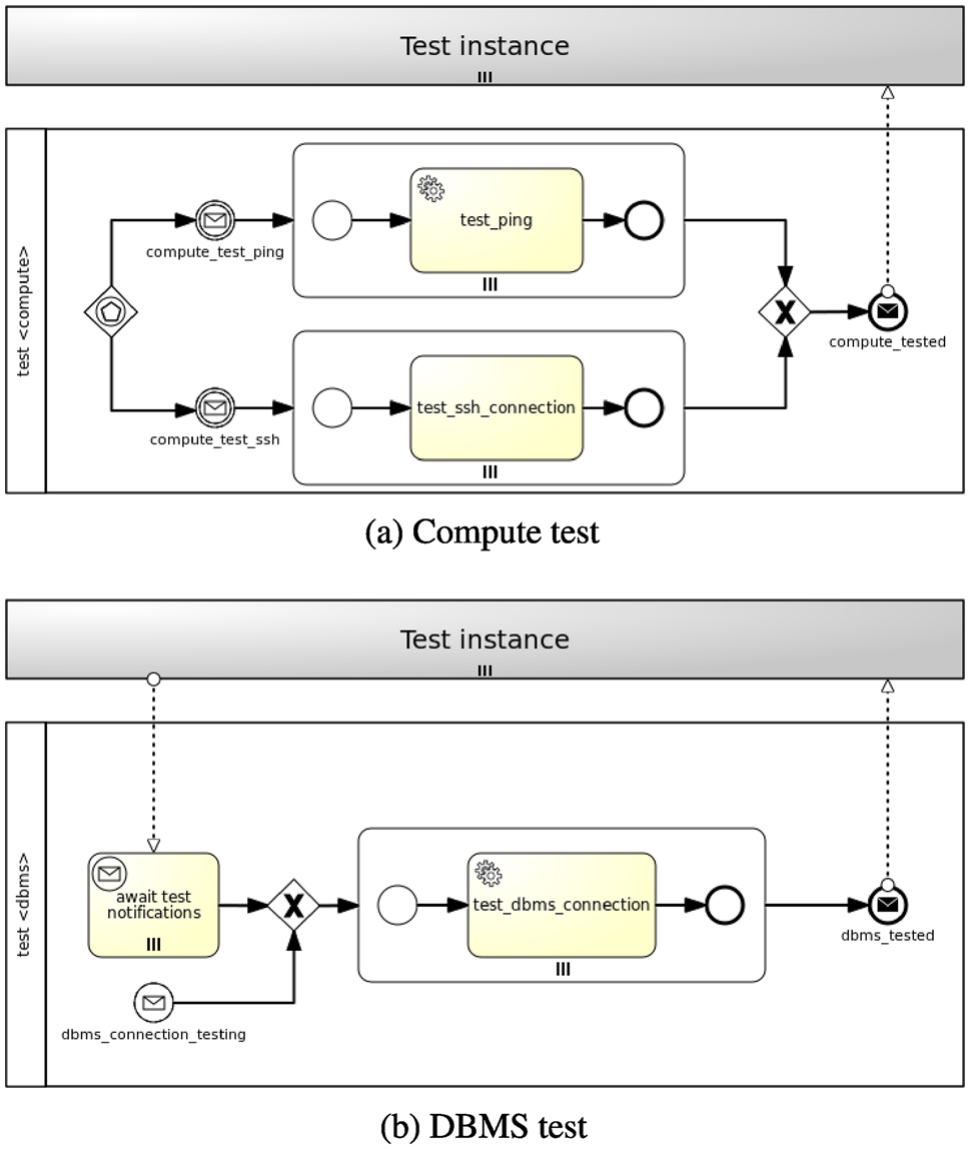
Illustrative Testing workflows for a WordPress scenario

Figure 12 shows exemplifying Testing workflows for the *mysql_server* and *app_server* nodes (Fig. 12a) and the *mysql_dbms* node (Fig. 12b) in the motivation scenario. In contrast with the strategy for the generation of Backup workflows, the strategy of generation of Testing workflows can take node dependencies into account. In particular, a distinction is made between nodes having no dependencies, such as *Compute* nodes, and nodes having dependencies, such as *DBMS* nodes.

In the first case (see Fig. 12a), given a *TestableCompute* policy targeting a *Compute* node, two paths are generated where either the *compute_test_ping* message intermediate event or the *compute_test_ssh* message intermediate event, coming from the TestCompute interface notifications, triggers the workflow instantiation by means of an event-based gateway. Regardless of the path being activated, a parallel multi-instance subprocess is then instantiated depending on the number of target nodes the policy is applied to. In this case there are two target nodes (i.e. *mysql_server* and *app_server*). Either the *test_ping* service task or the *test_ssh_connection* service task executes the action of the policy trigger, which enacts the actual Compute testing. Finally, the *compute_tested* message end event marks the end of the workflow.

In the second case (see Fig. 12b), given a *TestableDBMS* policy targeting a *DBMS* node, a new workflow is generated where either the *dbms_connection_testing* message start event, coming from the TestDBMS interface notifications, or the *await test notifications* parallel multi-instance receive task, waiting for notifications from node requirements under test (i.e. *mysql_server*), triggers the workflow instantiation. A parallel multi-instance subprocess is then activated depending on the number of target nodes the policy is applied to. In this case there is only one target node (i.e. *mysql_dbms*). The *test_dbms_connection* service task in the subprocess executes the action of the policy trigger, which enacts the actual DBMS testing. Finally, the *dbms_tested* message end event marks the end of the workflow.

### Update Feature

**Fig. 13:**
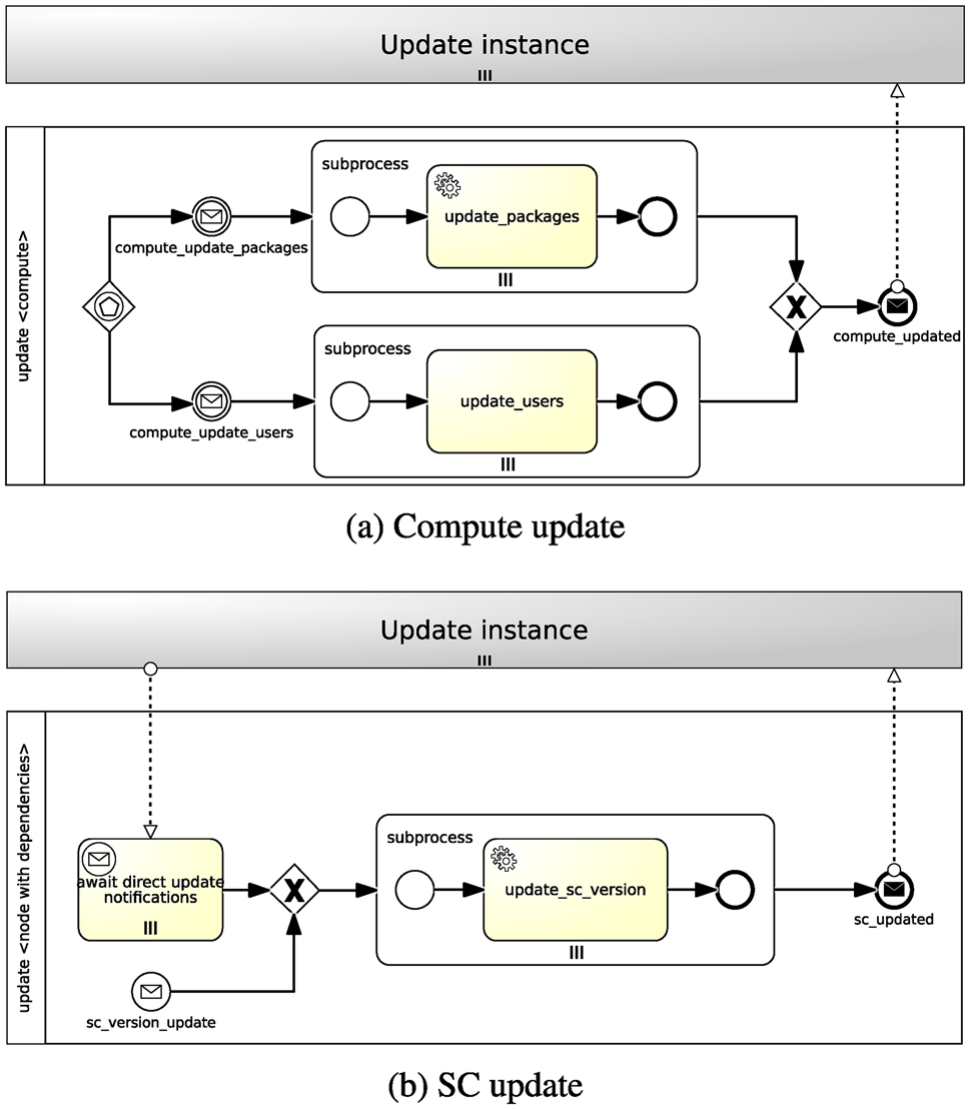
Illustrative Update workflows for a WordPress scenario

Figure 13 illustrates sample Update workflows for the *mysql_server* and *app_server* nodes (Fig. 13a) and the *php_sc* node (Fig. 13b) in the motivation scenario. Analogous to the generation of Testing workflows, the strategy for the generation of Update workflows can take node dependencies into account. Once again, there is a clear separation between nodes having no dependencies, such as *Compute* nodes, and nodes having dependencies, such as *SC* nodes.

With regard to nodes without dependencies (see Fig. 13a), considering an *UpdatableCompute* policy targeting a *Compute* node, two paths are generated where either the *compute_update_packages* message intermediate event or the *compute_update_users* message intermediate event, coming from the UpdateCompute interface notifications, triggers the workflow instantiation via an event-based gateway. No matter what path is activated, a parallel multi-instance subprocess is then instantiated based on the number of target nodes the policy is applied to. In this particular case there are two target nodes (i.e. *mysql_server* and *app_server*). A service task between the *update_packages* and the *update_users* executes the update action of the policy trigger. Finally, the *compute_updated* message end event marks the end of the workflow.

In case of nodes with dependencies (see Fig. 13b), given an *UpdatableSC* policy targeting an *SC* node, a new workflow is generated where one of the *await direct update notifications* parallel multi-instance receive task, waiting for notifications from direct node dependencies (i.e. *app_server*), and the *sc_version_update* message start event, coming from the UpdateSC interface notifications, triggers the workflow instantiation. A parallel multi-instance subprocess is then activated depending on the number of target nodes the policy is applied to. In this specific case there is only one target node (i.e. *php_sc*). The *update_sc_version* service task in the subprocess executes the update action of the policy trigger. In conclusion, the *sc_updated* message end event marks the end of the workflow.

## Related Work

Policy-based automation and application management have witnessed a substantial increase in popularity in business-oriented and research projects [29]. This section contains a brief literature overview on application management based on policies, TOSCA or similar approaches.

In Waizenegger et al. [31] the authors presented Policy4TOSCA, a policy-based service provisioning approach for automatic processing of TOSCA policy definitions in a TOSCA runtime. Policies were used to formalise non-functional security requirements. Although policies can be easily reused in different topologies, policy definitions are based on the very first version of TOSCA. In Zimmermann et al. [34] the authors proposed a concept of Deployment Enforcement Rules to specify and automatically enforce reusable security requirements and restrictions for TOSCA-based deployment models. In summary, we can conclude that these approaches mostly utilised policies to put security requirements into effect. However, they could be theoretically adapted to match different kinds of requirements as well.

In Alexander et al. [1] the authors presented TOSCAMP, an end-to-end cloud orchestration solution based on TOSCA and CAMP, which allows for multi-cloud application orchestration through declarative policies. In Di Modica et al. [12] the authors introduced a policy-based deployment in hybrid and multi-cloud environments. Policies were used to address non-functional requirements (e.g., security, geolocation). Even though both works leveraged policies for application orchestration on multi-cloud environments, no holistic management was considered.

In Pierantoni et al. [27] the authors introduced MiCADO, a cloud orchestration framework to deploy and manage TOSCA-based applications in the cloud. An extensible set of TOSCA policies was also elaborated to manage application deployment, performance, scalability and security requirements. In Caballer et al. [6] the authors presented INDIGO-DataCloud, a cloud orchestration platform to orchestrate TOSCA-based applications with complex topologies and operational requirements across heterogeneous cloud infrastructures. In Kumara et al. [20] the authors proposed the SODALITE platform to support the deployment, execution, monitoring and policy-based runtime adaptation of TOSCA-based applications on heterogeneous cloud-edge infrastructures. In Cankar et al. [10] the authors presented xOpera, an orchestrator of for enacting application deployment and autoscaling in a policy-based fashion by means of TOSCA templates. All the aforementioned works dealt with application deployment and policy-based management. Nevertheless, the main focus was on non-functional requirements, with no mention of holistic management.

In Wurster et al. [33] the authors presented a modelling concept to annotate a deployment model with automatic deployment tests. Limitations of the proposal include the fact that the deployment system must provide a basic set of plugins and be extensible with custom plugins from arbitrary sources. In addition, the approach features no other holistic management except testing. In Harzenetter et al. [18] the authors introduced an approach to automatically terminate an application in its current state and restart it in the same state again. Limitations of the proposal include the assumption that a stateful component type must be annotated to indicate that it will hold a state between requests. Besides, the approach features no other holistic management except state backup and recovery. In Harzenetter et al. [17, 19] the authors proposed a concept for the automatic generation of executable management workflows based on either an application deployment model or a running application. The modelled components are enriched with component-specific management operations and a management workflow gets generated to orchestrate such operations. However, the approach suffers from some limitations. To provide new management features, on the one hand, the workflow generation must be extended with a corresponding plugin and, on the other one, domain experts have to implement management operations in new component types.

In conclusion, the general picture emerging from the most recent academic research is that the majority of literature works centred around a specific management feature: either testing (i.e. [33]) or backup (i.e. [18]). On the other hand, a couple of works (i.e. [17, 19] integrated both management operations. Despite their similarities, our work differs from the aforementioned ones because it allows to:

- Utilise the update feature besides testing and backup ones;
- Provide a general holistic approach based on a TOSCA-compliant extension;
- Integrate supplementary management features without requiring additional components (e.g. plugins) installed on the framework.

## Conclusion

In a business scenario where resources and services are supplied by multiple cloud providers, one of the deciding factors for establishing competitive advantage is the automated provisioning and management of complex cloud applications. As a consequence, a number of orchestration frameworks have appeared to simplify the entire life-cycle management of cloud applications. Nevertheless, holistic application management involving multiple components distributed over heterogeneous environments is still an uncovered issue.

In this paper, we proposed a concept to automatically generate holistic application management workflows by enriching TOSCA application models with a combination of TOSCA interfaces, node types and policy types. The main steps of the process, namely the application model description and the management workflow generation, were thoroughly investigated. A prototype implementation of the framework was developed and a case study was also discussed to corroborate the practical feasibility of the approach.

In the future, the presented approach will be extended by following different research directions. Besides backup, testing and update, other management features will be investigated and integrated into the framework. Additional strategies for workflow generation will also be explored. Ultimately, supplementary case studies will be considered for further experimental validation.

## Data Availability

Data sharing not applicable to this article as no datasets were generated or analysed during the current study.
